# Association between simple renal cyst and kidney damage in a Chinese cohort study

**DOI:** 10.1080/0886022X.2019.1632718

**Published:** 2019-07-08

**Authors:** Juan Chen, Xiaojing Ma, Dongmei Xu, Wei Cao, Xianglei Kong

**Affiliations:** a Department of Nephrology, Shandong Provincial Qianfoshan Hospital, The First Hospital Affiliated with Shandong First Medical University, Jinan, China;; b Health Screening Center, Shandong Provincial Qianfoshan Hospital, The First Hospital Affiliated with Shandong First Medical University, Jinan, China

**Keywords:** Simple renal cyst, kidney disease, proteinuria, estimated glomerular filtration rate

## Abstract

**Background:** The presence of simple renal cyst (SRC) has been associated to renal dysfunction, but the results were inconsistent. Accordingly, we conducted a longitudinal cohort study to explore the association between SRC and kidney damage.

**Methods:** A total of 4274 adults (aged 45.4 ± 13.6 years) without chronic kidney disease at baseline were enrolled in 2008. SRC was assessed by ultrasonography. Logistic regression analysis were applied to explore the relationships between SRC and indicators of kidney damage (proteinuria and renal insufficiency), and also with relatively rapid decline in renal function (defined as the lowest quartile of △eGFR).

**Results:** During 5 years of follow-up, participants in the SRC group had higher incidence of proteinuria (5.2% versus 2.4%, *p* = 0.004) and renal insufficiency (3.8% versus 0.97%, *p* < 0.001) compared with control group. SRC was correlated with proteinuria (OR 2.24; 95% CI 1.34–3.75) and renal insufficiency (OR 4.0; 95% CI 2.11–7.58) in univariable analysis, despite that the correlation was not significant after adjusted for traditional kidney disease risk factors. Furthermore, after adjusted for potential confounders, maximum diameter of the cyst (≥2.2 cm) was significantly associated with rapid decline in renal function (OR 2.19; 95% CI 1.24–3.87).

**Conclusions:** Participants with SRC may be associated with higher incidence of proteinuria and renal insufficiency. This relationship may be obscured by age and other traditional risk factors. Higher diameter of the cysts contributed to more rapid decline in renal function of SRC participants.

## Introduction

Simple renal cysts (SRC) are common in the aging adult population. The availability and use of abdominal diagnostic ultrasonography and computed tomography have led to the frequent detection of asymptomatic renal cysts. The prevalence of SRC was found to be 7–10% depending on the study population and the methods, and most studies reported an increasing prevalence with increasing age [1,2]. Other factors that have been linked to the development of SRCs include male gender, hypertension, and renal dysfunction [3,4]. Since the diameter of a renal cyst may increase by 5% annually, the diameter of the cyst may increase by 1.6 times in 10 years [5]. In clinical practice, the nonhereditary forms of cystic lesions have received less attention except for SRC becoming symptomatic and presenting with abdominal discomfort, flank pain, or hematuria as a result of complications or a cyst expansion [6]. Nevertheless, recent evidence suggested additional linkages and problems about SRC such as the association with hypertension, kidney size, renal function, and the early and long-term allograft function [2,3,7–10].

Under normal physiologic condition, glomerular filtration rate (GFR) declines with aging by approximately 0.8 mL/min/1.73 m^2^ annually [11]. A decreased GFR is a risk factor of cardiovascular disease (CVD) [12,13] and associated with CVD mortality and morbidity in high-risk patients [14,15] and the general population [16]. Early and progressive decline in GFR, is thought to be an early marker of progressive kidney disease. Rapid GFR decline has also been shown to be associated with CVD and all-cause mortality [17]. Previous cross-sectional observational studies demonstrated a correlation between SRC and renal injury (proteinuria or renal insufficiency) [7,18]. However, controversy remains regarding the role and possible mechanisms relating these factors to the development of renal cysts [9]. Furthermore, cross-sectional studies cannot show the causal relationship between SRC and renal injury, and there has been a lack of prospective cohort study, especially in Chinese population. Therefore, we conducted this large longitudinal cohort study, aiming to investigate the prevalence of SRC in Chinese urban population without overt renal disease and explore the causal relationship between SRC and renal injury.

## Methods

### Study population

This study was a single-center retrospective cohort study. We analyzed the medical records of study subjects from the database who underwent annual medical examination in a large tertiary-care university hospital between 2008 and 2013. The participants were from all over Jinan and received a regular paid health examination. Participants with proteinuria and with decreased eGFR (<60 mL/min/1.73 m^2^) in the baseline were excluded. Patients with malignant disease, polycystic kidney, nephrolith, solitary kidney, heart failure, severe liver disease, infection disease, and pregnancy were also excluded. In 2008, a total of 4274 adults with whole medical records were enrolled and they were followed-up for 5 years. The ethics committee of Qianfoshan Hospital approved the study (2016-S001). All participants gave written informed consent prior to data collection. The study was fully complied with Helsinki regulation on human studies.

### Blood biochemistry measurements and biometric parameters at baseline

Blood was collected by means of venepuncture after an overnight fast of at least 10 h. Serum creatinine was measured by means of using the Roche enzymatic method on an automatic biochemistry analyzer (Roche P Modular with Roche Creatininase Plus Assay, Hoffman-La Roche, Ltd., www.roche.com). eGFR was calculated using the Chronic Kidney Disease Epidemiology Collaboration (CKD-EPI) two-level race equation [19]. Protein in urine was measured on a morning urine sample using an immediate semi-quantitative urine protein dipstick test and graded as negative, trace, 1+, 2+, 3+, or 4+. Subjects were considered to have proteinuria if their urinalysis showed ≥1+. Participants with pyuria were excluded from the analysis of proteinuria due to concern of urinary tract infection. Women during menstruation were asked to receive urine routine test 3 days after menstruation. Renal insufficiency was defined as the eGFR <60 mL/min/1.73 m^2^. Hemoglobin, urea nitrogen, fasting blood glucose, serum uric acid, serum total cholesterol (TC), and triglycerides (TG) were also measured by the automatic biochemistry analyzer.

Sociodemographic characteristics, health history (e.g. hypertension, diabetes), and lifestyle behaviors were obtained through questionnaire. Diabetes was defined as fasting blood glucose ≥7.0 mmol/L or the use of hypoglycemic agents or self-reported history of diabetes. Hypertension was defined as systolic blood pressure of more than 140 mmHg or diastolic blood pressure of more than 90 mm Hg, or both, or patients already being prescribed by antihypertensive medicaments. The body mass index (BMI) was calculated from body weight measured with light clothing to the nearest 0.1 kg and body height without shoes to the nearest 0.5 cm.

Experienced doctors of color Doppler ultrasound diagnostic device performed the abdominal ultrasound using a 3.5 or 5 MHz transducer to diagnose the SRC [8,9]. The SRC was defined as the anechoic lesion with homogeneity, water content, and a sharp interface to the adjacent renal parenchyma that did not have wall thickening, calcification, or enhancement on ultrasonography. We excluded patients with congenital renal cystic diseases. Renal cysts were classified into different groups based on the characteristics of the cysts, and the multiple cysts mean more than two cysts, the maximum diameter of cyst and the location of cyst were related to one or two kidneys.

All investigators have completed a training program of the methods and procedures of the study.

### Indicators of kidney damage

Proteinuria and eGFRs were reevaluated using the same strategy after 5 years follow-up. Renal insufficiency was defined as the eGFR <60 mL/min/1.73 m^2^. Decline in renal function (evaluated by the change in eGFR, △eGFR = 2013 eGFR-baseline eGFR) was calculated. Relatively rapid decline in renal function was defined as the lowest quartile of △eGFR. The 25, 50, and 75 percentiles of △eGFR for total participants was −7.04, −1.88, and 4.1 mL/min/1.73 m^2^, respectively. The 25, 50, and 75 percentiles of △eGFR for SRC subgroup was −6.41, −1.89, 3.93 mL/min/1.73 m^2^, respectively.

### Statistical analysis

Data were presented as proportions for categorical variables and mean ± SD or median [interquartile range (IQR)] for continuous variables. The significance of differences in continuous variables between SRC and the control groups were tested using independent-samples *t* test. The difference in the distribution of categorical variables was tested using Chi-square test. Univariate and multivariate logistic regression analysis were used to estimate risk factors associated with indicators of kidney damage. Independent variables included age, sex, hypertension, diabetes, smoking, drinking, uric acid, total cholesterol, triglycerides, BMI, and SRC.

The upper quartile of maximum diameter of cysts was also used as a categorical dependent variable for analysis, in comparison with the lower three quartiles. The 25, 50, and 75 percentiles of maximum diameter of renal cysts were 1.2, 1.6, and 2.2 cm, respectively. In subgroup analysis, only participants with SRC were included (*n* = 346). Logistic regression model was used to estimate the associations between different indicators of SRC and relatively rapid decline in renal function.

Crude and adjusted odds ratios (ORs) with 95% confidence interval (CI) were reported. All analyses were performed by SPSS statistical package, version 16.0 (SPSS, Inc., Chicago, IL). All *p* values are two tailed. *p* Values of less than 0.05 is considered statistically significant.

## Results

### Baseline characteristics of participants with SRC

Among 4274 participants in the study, the mean age was 45.4 ± 13.6 years (range 18–84 years), and 67.1% of them were males. The prevalence of SRC was 8.0% in the population. The baseline eGFR were 98.1 ± 14.7 mL/min/1.73 m^2^. Baseline characteristics of the participants stratified according to the SRC group were shown in Table 1. The average age of SRC group was significantly higher than the control group (54.6 versus 44.6 years; *p* < 0.001). Besides, the proportion of male was significant higher in SRC group (54.6 versus 44.6%; *p* < 0.001). Prevalence of hypertension and diabetes were also higher in SRC group than control group. The mean value of BMI, hemoglobin, uric acid, total cholesterol, and triglyceride were also significantly higher in SRC group. The mean eGFR of SRC group was lower than the group without SRC in baseline (*p* < 0.001).

**Table 1.** Clinical characteristics of participants stratified according to the renal cyst.

	Total	Renal cyst group	Control group	*p* Value
Number	4274	346	3928	/
Age (years)	45.4 ± 13.6	54.6 ± 13.6	44.6 ± 13.3	<0.001
Male (*n*, %)	2866 (67.1)	274 (79.2)	2592 (66.0)	<0.001
BMI (kg/m^2^)	24.7 ± 3.4	25.5 ± 3.2	24.7 ± 3.4	<0.001
Smoking (*n*, %)	766 (17.9)	76 (22.0)	690 (17.6)	0.048
Habitual drinking (*n*, %)	1342 (31.4)	125 (36.1)	1217 (31.0)	0.05
Hypertension (*n*, %)	885 (20.7)	106 (30.6)	779 (19.8)	<0.001
Diabetes (*n*, %)	409 (9.6)	51 (14.7)	358 (9.1)	0.001
Blood glucose (mmol/L)	5.72 (5.38–6.10)	5.91 (5.55–6.39)	5.71 (5.36–6.10)	<0.001
SBP (mmHg)	123.8 ± 17.9	130.0 ± 18.5	123.3 ± 17.8	<0.001
DBP (mmHg)	78.2 ± 11.7	81.9 ± 11.5	77.9 ± 11.6	<0.001
Hemoglobin (g/L)	143.3 ± 14.8	145.1 ± 13.1	143.2 ± 15.0	0.04
BUN (mmol/L)	4.8 ± 1.2	5.0 ± 1.3	4.8 ± 1.2	<0.001
Serum creatinine (μmol/L)	76.9 ± 12.4	79.7 ± 11.6	76.6 ± 12.5	<0.001
Uric acid (mg/dL)	5.3 ± 1.3	5.6 ± 1.2	5.3 ± 1.3	<0.001
Total cholesterol (mmol/L)	5.06 (4.47–5.73)	5.23 (4.63–5.89)	5.05 (4.46–5.72)	<0.001
Triglycerides (mmol/L)	1.12 (0.78–1.67)	1.27 (0.91–1.71)	1.11 (0.78–1.67)	<0.001
Baseline eGFR (mL/min/1.73 m^2^)	98.1 ± 14.7	90.6 ± 14.1	98.1 ± 14.3	<0.001

Abbreviation: BMI: body mass index; SBP: systolic blood pressure; DBP: diastolic blood pressure; BUN: urea nitrogen; eGFR: estimated glomerular filtration rate.

### SRC and renal injury incidence

During 5 years of follow-up, 112 (2.6%) patients developed proteinuria and 51 (1.2%) patients developed renal insufficiency. Participants in the SRC group had higher percentage of proteinuria (5.2% versus 2.4%; *p* = 0.004) and renal insufficiency (3.8% versus 0.97%; *p* < 0.001) compared with control group. There were no statistically significant difference in △eGFR in individuals with or without renal cyst (−1.59 ± 10.1 versus −1.39 ± 10.1 mL/min/1.73 m^2^) (Table 2).

**Table 2.** Outcomes of participants after 5-years follow-up stratified according to the renal cyst.

	Renal cyst group	Control group	*p* Value
Follow-up eGFR (mL/min/1.73 m^2^)	89.0 ± 15.5	96.7 ± 14.7	<0.001
△eGFR (mL/min/1.73 m^2^)	−1.59 ± 10.1	−1.39 ± 10.1	0.73
Proteinuria (*n*, %)	18 (5.2)	94 (2.4)	0.004
Renal insufficiency (*n*, %)	13 (3.8)	38 (0.97)	<0.001
Relatively rapid decline in renal function	78 (22.5)	1000 (25.5)	0.25

Abbreviation: eGFR: estimated glomerular filtration rate; △eGFR = follow-up eGFR- baseline eGFR.

### SRC and proteinuria

We conducted logistic regression to evaluate the relationship between SRC and proteinuria incidence. In univariate analysis, SRC were independently associated with proteinuria (OR 2.24; 95% CI 1.34–3.75). In subgroup analysis, multiple cysts (≥2 cysts) were significantly associated with higher proteinuria incidence (OR 2.73; 95% CI 1.02–7.33). Bilateral cysts (OR 2.18; 95% CI 0.68–6.97) and maximum diameter of cysts ≥2.2 cm (OR 2.07; 95% CI 0.78–5.54) were also positively related with proteinuria incidence, although statistics insignificant. After adjusted for age, sex, hypertension, and diabetes, the OR between SRC and proteinuria was 1.55 (95% CI 0.89–2.71). ORs of multiple cysts, bilateral cysts, and maximum diameter were 1.76 (95% CI 0.59–5.23), 1.32 (95% CI 0.34–5.03), and 1.83 (95% CI 0.64–5.24), respectively (Table 3).

**Table 3.** Multivariate logistic regression analysis for association between renal cyst with indicators of kidney damage.

Participants	Crude OR (95% CI)	Age- and sex-adjusted OR (95% CI)	Multivariable adjusted OR1* (95% CI)
Proteinuria (*n* = 112)			
Renal cyst	2.24 (1.34–3.75)	1.54 (0.89–2.63)	1.55 (0.89–2.71)
Subgroup^‡^			
Bilateral	2.18 (0.68–6.97)	1.84 (0.56–5.98)	1.32 (0.34–5.03)
Multiple cysts (≥2 cysts)	2.73 (1.02–7.33)	2.20 (0.80–6.07)	1.76 (0.59–5.23)
Maximum diameter (≥2.2 cm)	2.07 (0.78–5.54)	1.81 (0.65–5.01)	1.83 (0.64–5.24)
Renal insufficiency (*n* = 51)			
Renal cyst	4.0 (2.11–7.58)	1.70 (0.86–3.33)	1.55 (0.77–3.13)
Subgroup^‡^			
Bilateral	1.33 (0.29–6.23)	0.95 (0.19–4.67)	0.91 (0.18–4.67)
Multiple cysts (≥2 cysts)	2.63 (0.83–8.30)	1.64 (0.49–5.47)	1.72 (0.49–6.06)
Maximum diameter (≥2.2 cm)	3.88 (1.27–11.89)	2.77 (0.86–8.86)	3.14 (0.92–10.75)

Abbreviation: OR: odds ratio; CI: confidence interval; *OR1 was adjusted for age, sex, hypertension, diabetes. The 25, 50 and 75 percentile of maximum diameter of renal cysts were 1.2, 1.6 and 2.2 cm, respectively. Subgroup^‡^, included 346 participants with simple renal cysts.

### SRC and renal insufficiency

Logistic regression was conducted to analyze the relationship between SRC and renal insufficiency. In univariate analysis, SRC were independently associated with renal insufficiency (OR 4.0; 95% CI 2.11–7.58). In subgroup analysis, maximum diameter were significantly associated with higher renal insufficiency incidence (OR 3.88; 95% CI 1.27–11.89). Bilateral cysts (OR 1.33; 95% CI 0.29–6.23) and multiple cysts (OR 2.63; 95% CI 0.83–8.30) were also positively related with renal insufficiency, although statistics insignificant. After adjusted for age, sex, hypertension, and diabetes, the OR between SRC and renal insufficiency was 1.55 (95% CI 0.77–3.13). ORs of multiple cysts and maximum diameter of SRC with renal insufficiency were 1.72 (95% CI 0.49–6.06) and 3.14 (95% CI 0.92–10.75), respectively (Table 3).

### SRC and rapid renal function decline

In this part, risk for rapid decline in renal function was analyzed, and results showed that no significant difference existed between SRC group and control group (OR 0.94; 95% CI 0.72–1.24). In subgroup analysis, only patients with SRC were included (*n* = 346). The patients were divided by the SRC characters, such as bilateral cysts, cysts number ≥2, and maximum diameter of the cyst ≥2.2 cm independently. Univariable logistic regression showed that maximum diameter of the cyst was significantly correlated with more rapid decline in renal function, and the OR was 1.90 (95% CI 1.11–3.25). After adjusted for age, sex, hypertension, diabetes, smoking, drinking, uric acid, total cholesterol, triglycerides, and BMI, the correlation was also robust (OR 2.19; 95% CI 1.24–3.87), Table 4.

**Table 4.** Multivariate logistic regression analysis for association between renal cyst with relatively rapid decline in renal function.

Participants	Crude OR (95% CI)	Age- and sex-adjusted OR* (95% CI)	Multivariable adjusted OR^†^ (95% CI)
Renal cyst	0.85 (0.66–1.11)	0.97 (0.74–1.27)	0.94 (0.72–1.24)
Subgroup^‡^			
Bilateral	0.57 (0.24–1.33)	0.58 (0.25–1.37)	0.43 (0.17–1.10)
Multiple cysts (≥2 cysts)	0.89 (0.48–1.66)	0.93 (0.50–1.76)	0.80 (0.41–1.58)
Maximum diameter (≥2.2 cm)	1.90 (1.11–3.25)	2.09 (1.20–3.62)	2.19 (1.24–3.87)

Abbreviation: OR: odds ratio; CI: confidence interval.

*OR was adjusted for age and sex.

^†^OR was adjusted for age, sex, hypertension, diabetes, smoking, habitual drinking, uric acid, total cholesterol, triglycerides and baseline BMI.

Subgroup^‡^, included 346 participants with simple renal cysts. Relatively rapid decline in renal function was defined as the lowest quartile of △eGFR. The 25, 50 and 75 percentile of △eGFR for total participants was −7.04, −1.88 and 4.1 mL/min/1.73 m^2^, respectively. The 25, 50 and 75 percentile of △eGFR for SRC subgroup was −6.41, −1.89, 3.93 mL/min/1.73 m^2^, respectively.

## Discussion

The results about the associations between SRC and kidney damage were still controversial, and some previous reports showed contrary results [9,20,21]. Our previous cross-sectional observational studies demonstrated that participants with one or more cysts had higher percentage of proteinuria and decreased eGFR (DeGFR). Furthermore, SRC was correlated with proteinuria (OR 1.59; 95% CI 1.30–1.95) and DeGFR (OR 1.97; 95% CI 1.56–2.47) after adjusted for potential confounders. Our results also demonstrated that maximum diameter (per 1 cm increase), bilateral location, and multiple cysts significantly correlated with DeGFR [18]. However, cross-sectional study cannot explore the causal relationship or provide an insight into the mechanisms responsible for the observed associations. Thus, we conducted this retrospective cohort study, with a long-term observation in China, to identify a causal correlation between SCR and renal injury.

Our participants were composed of people who underwent routine health checkup. Baseline characteristics of the participants showed that individuals with SRC were significantly older than those without cyst, confirming the high prevalence of SRCs in elderly individuals. We also found a significant predominance of male sex in occurrence of SRC. Besides, the proportion of hypertension and diabetes were higher in SRC group. The causal and temporal relationship between SRC with hypertension remains controversial. Serum creatinine, atherosclerotic diseases, and hypertension are among the main implicated risk factors in the occurrence of SRC.

We retrospectively analyzed the influence of SRC on kidney damage after adjusted by potential confounding effects of age, gender, hypertension, and diabetes. Study subjects were also grouped by the characteristics of cyst, such as the number and the diameter, to determine the association between these features and kidney damage. The results demonstrated that during 5 years of follow-up, participants in the SRC group had higher incidence of proteinuria and renal insufficiency compared with control group, although not statistic significant after adjusted by traditional kidney disease risk factors. Previous observational studies with positive results were almost cross-sectional, which cannot identify a causal relationship. Ai et al. reported that presence of SRC was associated with reduced renal function in patients younger than 60-year-old [7]. This relationship may be obscured by the reduced renal function and the high incidence of cysts in older patients. This would partly explain why the OR in our study was insignificant after adjusted by age, sex, hypertension, and diabetes.

After adjusted by other confounders, our results showed that maximum diameter of the cyst (≥2.2 cm versus <2.2 cm) was associated with relatively rapid decline in renal function (OR 2.19;95% CI 1.24–3.87). The result demonstrated that larger diameter of the cysts might contribute more to the decline in renal function among SRC patients. Some pathophysiological mechanisms might explain our results. Renal cyst development occurs as the proliferation of epithelial cells with tubular ectasia, saccular dilatation, and fluid collection. It is postulated that tubular obstruction and ischemia contributed to the formation of cysts, which occur more often in the cortex area. Morphologically, SRCs are oval to round. They may be single or multiple. Simple cysts can increase in size over time by 5% annually [5]. The increased incidence of cysts may be a manifestation of the progressive nephron loss that occurs with aging [22]. Primary hypertension is closely related to the reduced number of nephrons [23]. Renal cyst expansion may cause local ischemia or renal arterial compression, and this may activate the rennin-angiotensin system [24,25], and plays a pivotal role in the development of hypertension [26], which was well-known risk factors for kidney damage. Copeptin, a marker of vasopressin, was associated with kidney length and prevalence of SRC in a population-based study [27]. In healthy rats, increased vasopressin had a proalbuminuric effect [28]. Moreover, in the population-based studies, vasopressin also might be involved in the development of proteinuria [29,30]. However, there has been some evidence that renal cysts in patients aged <50 years progressed more rapidly than older patients. The only predicted factor for the growth rates was younger age (<50 years) [31,32]. However, pathogenesis of SRC is still unsubstantiated. The connection of renovascular and arterial pathologies with SRC can merely be coincidental to age-related changes in renal tubules and ducts. While origins and evolution of SRC still need to be explored, the only established link is senility [32].

However, this study also has limitations that deserve mention. First, this was a retrospective study in single center and was implemented not based on a community-based screening. Therefore, selecting bias in the study limited the extension of the results. Second, we used a single morning sport urine sample to assess proteinuria, instead of urinary albumin-to-creatinine ratio which would be more preferable. And single measurements of serum creatinine at baseline and serum creatinine after 5 years could have resulted in the misclassification of exposure, confounders, and outcomes. Third, we used the existing ultrasound results of health examination so we could not correct the intrapersonal or interpersonal variability to detect and localize the SRC or to measure the maximum diameter of cyst in charge of routine health checkup. Finally, although renal ultrasonography is a reliable tool for estimating SRC, it is not as sensitive as computed tomography or magnetic resonance imaging to detect SRC, and very small cysts might have been missed.

In summary, SRC appeared to increase the incidence of renal injury. Furthermore, high diameter of the cysts might have contributed to more rapidly decline in renal function among SRC patients. However, our study was retrospective and cannot provide an insight into the mechanism responsible for the observed associations. Further prospective studies are needed to clarify whether intentional aspiration of cysts offers additional benefits to the renal, as well as the cardiovascular risk profile in the long term.
